# Structural white matter and functional connectivity alterations in patients with shoulder apprehension

**DOI:** 10.1038/srep42327

**Published:** 2017-02-08

**Authors:** Davide Zanchi, Gregory Cunningham, Alexandre Lädermann, Mehmet Ozturk, Pierre Hoffmeyer, Sven Haller

**Affiliations:** 1 Department of Psychiatry (UPK), University of Basel, Basel, Switzerland; 2 Division of Orthopaedic and Trauma Surgery, Department of Surgery, University Hospitals of Geneva, Switzerland; 3 Faculty of Medicine of the University of Geneva, Switzerland; 4 Division of Orthopaedic and Trauma Surgery, La Tour Hospital, Geneva, Switzerland; 5 Affidea Carouge Radiologic Diagnostic Center, Geneva, Switzerland; 6 Department of Surgical Sciences, Radiology, Uppsala University, Uppsala, Sweden; 7 Department of Neuroradiology, University Hospital Freiburg, Germany

## Abstract

Previous functional magnetic resonance imaging (fMRI) findings indicate that shoulder apprehension is more complex than a pure mechanical problem of the shoulder, showing a direct modification in functional brain networks associated with motor inhibition and emotional regulation. The current study extends these findings by investigating further structural alterations in patients with shoulder apprehension compared to controls. 14 aged patients with shoulder apprehension (27.3 ± 2.0 years) and 10 matched healthy controls (29.6 ± 1.3 years) underwent clinical and fMRI examination including fMRI and diffusion tensor imaging (DTI). Tract-based spatial statistics procedure was used to analyze white matter (WM) alterations. Functional images were analyzed investigating resting state network connectivity. DTI results were correlated with different shoulder clinical scores and functional connectivity networks. Fractional anisotropy (FA), representing white matter integrity, is increased in the left internal capsule and partially in the thalamus in patients compared to controls. Moreover, FA correlates negatively with simple shoulder test (SST) scores (*p* < .05) and positively with a functional connectivity network qualitatively replicating previous results (*p* < .01). This study extends previous findings, showing that in addition to functional changes, structural white matter changes are also present in patients with shoulder apprehension.

Anterior glenohumeral instability has been recognized as a common ailment for individuals of all demographics^1^. Its prevalence is approximately 1.7% of the population^2^. A common sign of this pathology is apprehension, defined by fear of imminent dislocation elicited when bringing the arm to 90° of abduction and 90° of external rotation^3^. This clinical condition can be associated to increased morbidity for the patient, long time away from work and a general decrease in life quality^4^,^5^.

Recent arising new interest in shoulder apprehension has helped disentangle its different cognitive and physical components combining functional magnetic resonance (fMRI) with clinical examination^6^. Previous fMRI findings have indeed indicated that apprehension is more complex than a purely mechanical problem of the shoulder, suggesting a direct implication of cerebral networks including motor, interoceptive awareness, and emotional regulation^6^,^7^. These results demonstrate functional differences at the brain level in patients with shoulder apprehension compared to normal population.

The current study extends these findings and assesses structural abnormalities in patients with shoulder apprehension using diffusion tensor imaging technique (DTI), an imaging technique to assess white matter (WM) microstructural changes, by showing the presence of structural alterations in WM in patients with shoulder apprehension. Moreover, we were able to link these structural modifications with functional changes identified in our previous investigation and with clinical scores measuring dimensions as shoulder performance in daily life.

## Results

### Analysis of Demographic data

The Mann-Whitney test, used to compare the participants age, was not significant, but it approached the significance level (*p* = .07).

### DTI results

**Figure 1:**
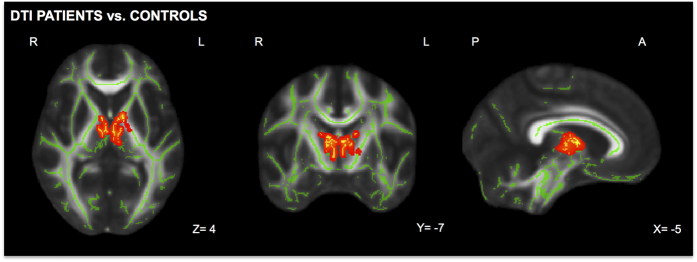
Significant white matter differences between patients with shoulder apprehension and controls. After permutation based non-parametric test, results of TBSS analyses revealed significant white matter FA increase (in red) in the left internal capsule and partially in the thalamus in patients compared to controls. Significant regions are shown at the threshold of P < 0.05 corrected for multiple comparisons using threshold-free cluster enhancement (TFCE).

Results from tract based spatial statistic (TBSS) analyses revealed a significant increase in fractional anisotropy (FA) in the left internal capsule and partially in the thalamus in patients compared to controls (Fig. 1). No significant results were found comparing patients with right-sided glenohumeral instability and left-sided glenohumeral instability.

### Correlation DTI results and clinical scores

**Figure 2:**
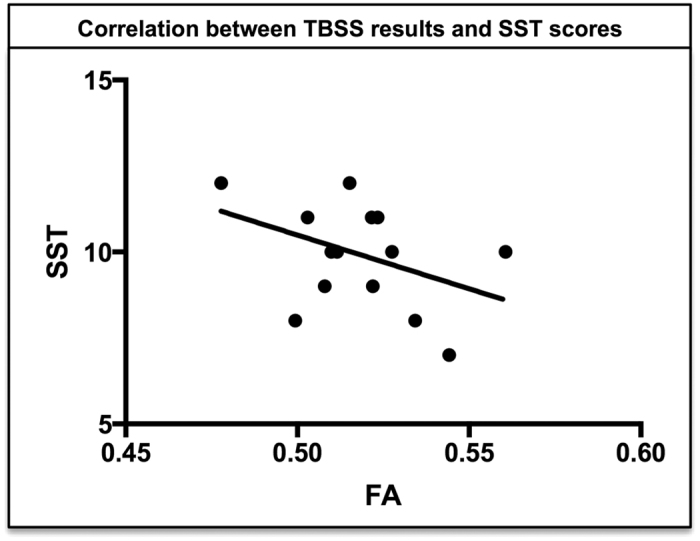
Correlations between TBSS results and clinical scores. In the patient group, significant negative correlations were found between TBSS results and SST scores (*P* < .05).

Significant negative correlations were found between TBSS results and Simple shoulder test (SST)^8^ scores (r = −.25, *p* < .05) (confidence intervals: −0.72, −.11) (Fig. 2). No significant correlations were found between TBSS results and the remaining tested scores: Rowe test^9^, Western Ontario shoulder instability (WOSI)^10^, Subjective Shoulder Value (SSV)^11^. Our results show that none of the confidence intervals after bootstrapping analyses included 0.

### FMRI results and correlation between structural and functional changes

**Figure 3:**
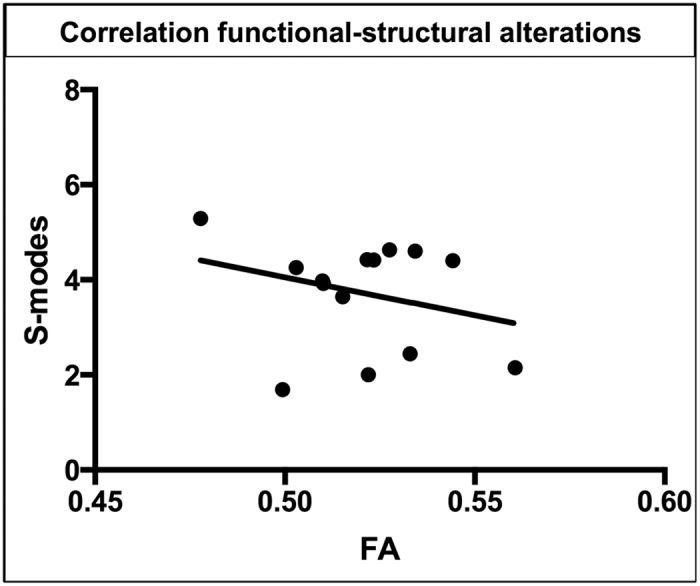
Correlation fMRI-DTI results. After Pearson correlations FA values of the left internal capsule and partially the thalamus of patients with shoulder apprehension negatively correlate (*P* < .01) with S-modes values of the identified ICA component.

After Tensor Independent component analyses (T-ICA), paired t-test analyses on S-modes values revealed significant differences (r = −.24, *p* < .05) (confidence intervals: −0.63, −16) between controls and patients in a brain network showing hypoactivations in the ventral anterior cingulate cortex, posterior cinculate and precuneus, and hyperactivation of anterior insula, motor and somatosensory cortex. These results qualitatively replicate the findings of our previous study^3^ (Supplementary Digital Content 1). Moreover, Pearson correlation showed a negative correlation between FA values of the left internal capsule and partial thalamus (TBSS results) with S-modes values of the identified ICA component (*p* < .01) (Fig. 3). Our results show that none of the confidence intervals after bootstrapping analyses included 0.

## Discussion

Our previous investigations showed global changes in functional cerebral networks in shoulder apprehension patients versus controls^3^. Moreover, we were able to link five established clinical scores of different dimensions associated to shoulder apprehension to functional imaging results in patients with a positive apprehension sign^6^. The current study extends our previous work on functional changes^6^ by demonstrating the presence of increased structural white matter connectivity in the left internal capsule and part of the thalamus in patients with shoulder apprehension compared to controls, suggesting structural neuronal plasticity. Furthermore, we were able to link our results to pain perception and shoulder performance measured by a clinical score (SST). Finally, we confirm our previous work by showing a significant correlation between the identified structural changes and functional connectivity alterations.

The most important finding of this study is the presence of increased WM connectivity in the left internal capsule and in the thalamus in patients with shoulder apprehension compared to controls. The internal capsule is a brain region part of the pyramidal tract where motor fibers travel to and from the cortex^12^,^13^. Increased FA in patients with shoulder apprehension suggests abnormal increased axonal integrity and therefore pathological structural plasticity due to the over-connection of WM fibers in the motor pathway. Differences in white matter between controls and patients are corroborated by a study by Lieberman *et al*.^14^, showing an increased WM connectivity in patients with chronic musculoskeletal pain compared to controls, using the same technique (TBSS). In line with these results, our work extends these previous findings showing an increase in subcortical FA during the condition of shoulder apprehension.

Our second finding relates to the negative correlation between subcortical WM alterations and the SST score that measures shoulder performance in daily activities. We interpret low SST scores in line with previous works^15^, suggesting that lower shoulder functioning in daily activities can be associated to shoulder apprehension due to the avoidance of certain movements. In our first neuroimaging work^3^, we demonstrated anxiety and avoidance of potentially harmful movements as cognitive conditions associated with shoulder apprehension. Our current investigation extends these findings, relating for the first time decreased shoulder performance to WM alterations during shoulder apprehension. The link between WM changes and avoidance of specific movements is confirmed by a study of Westlye *et al*.^16^ in which the authors link harm avoidance with increased WM microstructure. We conclude that shoulder apprehension is a pathological condition that can be associated to avoidance of specific movements; this leads to a decreased shoulder performance in daily activities that affects structural plasticity in the left internal capsule and partially in the thalamus.

Finally, the previous interpretations are confirmed also by the negative correlation between structural and functional changes in the brain of patients with shoulder apprehension. In our previous paper, in fact, we established a link between the measure of stability and mobility of the shoulder (clinical tests) and cognitive processes. Our previous study, investigating the neural bases of shoulder apprehension shows evidence of dynamic interaction between shoulder apprehension, and motor regulation, such that increased orbito-frontal cortex activity is related to a decreased activity in regions associated to shoulder apprehension, which in turn leads to a decrease in disability. From these results, more functional connectivity in frontal areas results in higher shoulder stability and movement allowance.

The correlation we found in the present work allows us to show how structural changes underlie functional changes. In particular, following the previous interpretation, the correlation between WM structural alterations and functional connectivity indicates that subcortical WM changes are linked to functional connectivity changes in shoulder apprehension networks.

These results demonstrate a continuum between our previous works^3^,^6^ and the following one, showing that shoulder apprehension, involving different physical and cognitive dimensions, leads to modifications of the whole brain architecture, including functional and structural plasticity alterations that lead to different shoulder performance in daily life. These findings strengthen our conclusion, showing that even if a successfully stabilized patient has a clinically stable shoulder, a persistent cerebral “scar” from apprehension can persist and impede a full recovery. This emphasizes the further need to extend investigations on the link between peripheral orthopedic pathologies and the central nervous system. These two systems are in fact interdependent and while it is accepted that the brain is in command of the joint, the use of neuroimaging in the exploration of orthopedic science is suggesting that the opposite seems to become more and more realistic.

### Limitations

Since apprehension involves both lateralized and non-lateralized cerebral zones, patient group selection had to be selective (same hand dominance): this study therefore did not include any left-handed patients. On the other hand, in our functional results, we observed highly significant brain functional network modifications related to apprehension. This indicates a strong effect of shoulder apprehension on cerebral networks. These results were not extensively discussed here as they resemble findings from previous studies^3^,^7^. Moreover, a limitation regarding the methods should be highlighted: we used DTI sequence with 30 diffusion directions. More directions can reveal more profound white matter changes in the patient group. This can explain also the absence of differences we found between patients with right and left- sided glenohumeral instability. In addition, the number of participants in each group was relatively small; therefore future studies should include larger study groups. Finally, pain and instability are very difficult to dissociate as apprehension is a complex condition that may be associated to many cognitive aspects such as anxiety, fear and pain. However, pain experienced by our patients is very weak (pain VAS ≤3) or absent (pain VAS = 0) and that none of the patients had pain during baseline nor during the MR imaging.

### Future directions

Another interesting aspect to further investigate will be the pre- versus post-surgical imaging and follow-up to evaluate the evolution of the cerebral modifications related to shoulder apprehension. Moreover as pointed already in our previous study^6^, other conditions such as knee, elbow or ankle instability should be assessed to test the hypothesis that apprehension-related modifications in cerebral neuronal networks are presumably generic and not limited only to shoulder apprehension. Finally new approaches to shoulder apprehension management, such as fear extinction or neurofeedback^17^ can be tested due to their promising results^18^.

## Conclusion

This study extends previous works^3^,^6^,^7^, showing that beside functional changes, WM changes are also present in patients with shoulder apprehension compared to controls affecting notably the motor tract in the internal capsule and bilateral thalamus. These structural alterations affect dimensions of shoulder apprehension as performance in daily life.

## Methods

The presented work is an additional and extended analysis of an fMRI dataset previously published by Cunningham *et al*.^6^.

### Participants

The protocol was approved by the University Hospitals and University of Geneva Ethical Committees and conducted in accordance with the principles of the Declaration of Helsinki. All experimental procedures were carried out in accordance with the approved guidelines. 38 subjects provided written informed consent prior to their participation. Both controls and patients were recruited at the University Hospital of Geneva (HUG), Switzerland. Inclusion criteria for the patient group were any patients presenting to a specialized shoulder surgery consultation with a history of traumatic anterior glenohumeral instability, a positive apprehension test, and radiologic evidence based on MR-arthrogram or CT scan. Patients with multi-directionnal, atraumatic or voluntary instability were not included in the study. Pain alone was not considered as a positive apprehension test. As stated by previous works, cutoff values for pain VAS are variable and adaptable in clinical practice^19^. For clarity reasons, we decided to use a trisection cutoff to interpret pain VAS scores (VAS 0-3: No or weak shoulder pain; VAS 4-6: Mild or modulate shoulder pain; VAS 7-10: Severe or strong shoulder pain) for inclusion and exclusion criteria^20^,^21^. Only patients showing no pain or weak pain (pain VAS scores ≤3) were included, patients with pain VAS scores >3 were excluded. Patients with pain at rest, or with an acute event of instability (within 6 previous months) were excluded. The control group consisted in healthy volunteers with no history of shoulder injury, instability, or hyperlaxity, the latter defined as more than 85° of external rotation elbow against waist^22^, or hyperabduction over 105°^23^. General exclusion criteria for both groups were: history of drug or alcohol abuse, major medical disorders or use of psychotropics, stimulants or β-blockers. The final sample included 14 male patients with shoulder apprehension (27.3 ± 2.0 years old) (10 right-sided and 4 left-sided glenohumeral instability, same hand dominance) and 10 healthy matched male controls (29.6 ± 1.3 years old).

### Clinical scores assessment

As described by Cunningham *et al*.^6^, prior to fMRI examination, patients were asked to fill five subjective functional scores frequently used in shoulder surgery to assess clinical dimensions associated to shoulder apprehension:

Pain Visual Analog Scale (VAS) was used to rate patients’ shoulder pain^24^ and to exclude patients with high pain (>3), Simple Shoulder Test (SST) to evaluate shoulder performance in daily activities^8^. Subjective Shoulder Value (SSV) is a test to assess the overall shoulder function^11^. ROWE score assesses shoulder stability and motion^9^. Western Ontario Shoulder Instability (WOSI) measures the degree of disability in activities of daily living^10^.

### FMRI and DTI parameters

All participants underwent a functional and structural MRI examination. The order of the scan included first a T1 sequence followed by a functional (EPI) sequence and finally by a diffusion tensor imaging (DTI) sequence.

Images were obtained using a 3T scanner (Trio; Siemens, Erlangen, Germany) with a standard 32-channel head-coil. The 3D T1-weighted structural scan presented the following parameters: 256 × 256 matrix size, 176 sections, 1 × 1 × 1 mm^3^, TE = 2.3 ms, TR = 2300 ms. The fMRI imaging of the whole brain was performed using echo planar imaging employing the following parameters: whole brain coverage, 96 × 96 matrix, TR = 2.5 s, TE = 30 ms, 39 slices, 148 repetitions. During the functional sequence cue videos showing daily activities that trigger shoulder apprehension were used as described previously^3^,^6^. Finally, the DTI scan was acquired with the following parameters: 30 diffusion directions *b* = 1000 s/mm^2^ isotropically distributed on a sphere, 1 reference *b* = 0 s/mm^2^ image with no diffusion weighting, 128 × 64 matrix, 2 × 2 × 2 mm voxel size, TE = 92 ms, TR = 9000 ms. For further details on the fMRI acquisition refer to Cunningham *et al*.^6^.

### Statistical Analysis

Statistical analyses were performed using GraphPad Prism (Version 6, GraphPad Software, San Diego, USA) and FMRIB Software Library (FSL) (Version 5.0.6, FMRIB, Oxford, UK).

### Analysis of Demographic data

After performing D’Agostino-Pearson omnibus test to check for normal distribution, we compared the participants’ age, non-normally distributed variable, using Mann-Whitney test.

### DTI analyses

The current structural analyses extend the functional analysis of our previous work^6^. Tract-based spatial statistics (TBSS) FSL procedure^25^ was used to analyze DTI images, adjusting the general linear model for age considered as non-explanatory variable.

TBSS normalizes individual brains and assesses alterations in regional white matter, which reflects axonal integrity. Threshold-free cluster enhancement (TFCE) multiple comparison correction was applied, considering corrected P values < 0.05 as significant.

### Correlations between DTI results and clinical scores

We tested correlations in the patient group between white matter alterations and clinical test scores. FA values extracted from the left internal capsule and the thalamus (TBSS results) and final scores of each test were submitted to Pearson correlation. FDR correction was performed for multiple comparisons. As sanity check bootstrapping analyses were performed using 5,000 bootstrap samples. Results were considered significant if 95% confidence intervals did not contain zero.

### FMRI analyses and correlation between structural and functional changes

The current analyses extends the functional analysis of our previous work^6^. In principle, multi-session tensor-independent component analyses was carried out in FSL using multi-session multivariate exploratory linear optimized decomposition into independent components (MELODIC)^26^ setting the number of components to 25. To test for group differences in brain network activations, the degrees of the connectivity strength (S-modes) were compared between groups using a 2-sample t-test with false discovery rate correction for multiple comparisons. To test if in the patients group structural changes were associated to functional changes, Pearson correlation analyses were performed between FA values from TBSS results and S-modes values of the identified independent component. Results were considered significant if 95% confidence intervals did not contain zero.

## Additional Information

**How to cite this article**: Zanchi, D. *et al*. Structural white matter and functional connectivity alterations in patients with shoulder apprehension. *Sci. Rep.* **7**, 42327; doi: 10.1038/srep42327 (2017).

**Publisher's note:** Springer Nature remains neutral with regard to jurisdictional claims in published maps and institutional affiliations.

## Supplementary Material

Supplemental Data
