# Supplementary material for: Structural white matter and functional connectivity alterations in patients with shoulder apprehension
Source: Sci Rep. 2017 Feb 8;7:42327. doi: 10.1038/srep42327 (PMC5296739; doi:10.1038/srep42327)
Supplement: Supplemental Data [file srep42327-s1.pdf]

**Title**
Structural white matter alterations in patients with shoulder apprehension.

**Short title**

Shoulder apprehension and brain structural changes.

**Authors**

Davide Zanchi[1], Gregory Cunningham[2], Alexandre Laedermann[2,4,7], Mehmet Ozturk[2], Pierre Hoffmeyer[2], Sven Haller[3,4,5,6]

**Affiliations**

**1** Department of Psychiatry (UPK), University of Basel, Basel, Switzerland
**2** Division of Orthopaedic and Trauma Surgery, Department of Surgery, University Hospitals of Geneva, Switzerland;
**3** Affidea Carouge Radiologic Diagnostic Center, Geneva, Switzerland
**4** Faculty of Medicine of the University of Geneva, Switzerland
**5** Department of Surgical Sciences, Radiology, Uppsala University, Uppsala, Sweden
**6** Department of Neuroradiology, University Hospital Freiburg, Germany
7 Division of Orthopaedic and Trauma Surgery, La Tour Hospital, Geneva, Switzerland

**Type of Manuscript**
Original manuscript

**Word count**
Original Manuscript: 2352
Abstract: 197
Figures: 3
Supplementary material: 1

**Topics**
Pain, white matter, internal capsule, fMRI, DTI, plasticity

**Corresponding author**

Davide Zanchi, PhD candidate.
Department of Neuropsychiatry,
University Pyschiatry Clinic
Wilhelm Klein-Strasse 27,
4012 Basel, Switzerland.
0041789084000
Davide.Zanchi@upkbs.ch

**SUPPLEMENTARY MATERIAL**

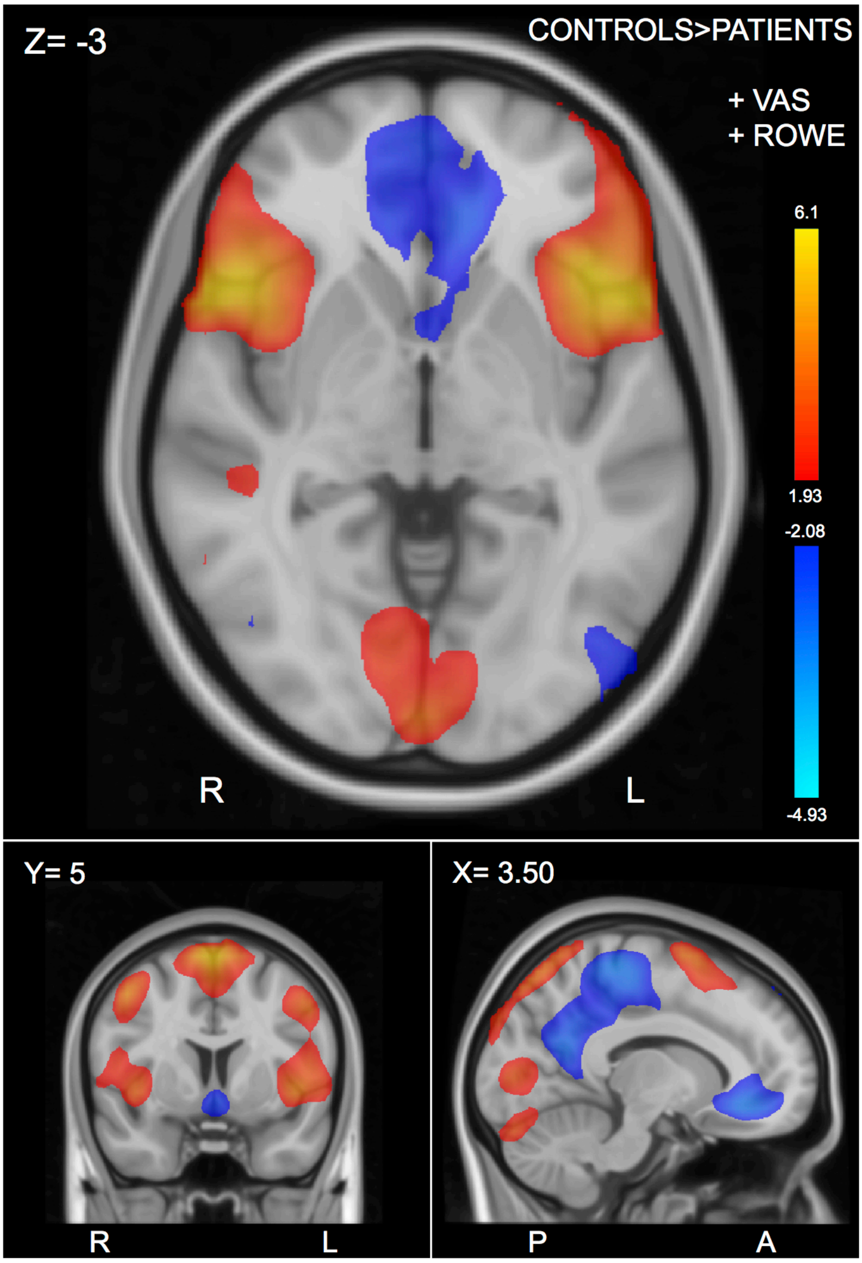


ICA results. After Tensor ICA, paired t-test analyses on S-modes values revealed significant differences (p<.01) between controls and patients in a brain network showing hypoactivations in the ventral anterior cingulate cortex, in the posterior cingulate and precuneous, and hyperactivation of anterior insula, motor and somatosensory cortex.
